# A Rare Case of Flavocoxid-Induced Pulmonary Fibrosis

**DOI:** 10.7759/cureus.9617

**Published:** 2020-08-08

**Authors:** Dimitrios Drekolias, Naga Vaishnavi Gadela, Philip Krupka, Jennifer Broess, Jason Jacob

**Affiliations:** 1 Internal Medicine, University of Connecticut, Farmington, USA; 2 Internal Medicine, Rush University, Chicago, USA; 3 Medicine, Hartford Hospital, Hartford, USA

**Keywords:** flavocoxid, limbrel, pulmonary fibrosis, hypersensitivity pneumonitis

## Abstract

Pulmonary fibrosis is a chronic progressive interstitial lung disease characterized by repetitive cycles of epithelial cell injury and dysregulated repair. Although most cases of pulmonary fibrosis are idiopathic, a detailed history that includes medications, comorbidities, tobacco use, environmental exposures, and family history should be taken to rule out secondary etiology. We present a case of flavocoxid-induced pulmonary toxicity which progressed from hypersensitivity pneumonitis to pulmonary fibrosis even after discontinuation of the offending drug.

## Introduction

Flavocoxid is a nonspecific cyclooxygenase and lipoxygenase inhibitor formerly used in the treatment of osteoarthritis that was recalled from the market in 2017 after reports of severe adverse drug reactions including hypersensitivity pneumonitis and liver toxicity emerged. It was initially marketed in 2004 as a plant-based prescription medical food that provided natural extracts such as catechin and baicalin. It was thought that flavocoxid had very few side effects, which were comparable to placebo, when used for treatment of mild to moderate osteoarthritis [1-2]. This was attributed to its unique mode of action, namely, the dual inhibition of cyclooxygenase and lipoxygenase as well as to its anti-inflammatory properties owing to its ability to absorb reactive oxygen species [3]. It was advertised as a medical food which is defined as a food which is formulated to be consumed or administered enterally under the supervision of a physician intended for the specific dietary management of a disease or a condition for which distinctive nutritional requirements are needed. Available literature suggested that these adverse effects were reversed following discontinuation of the drug; however, we present a case of irreversible pulmonary toxicity which, to the best of our knowledge, is secondary to the use of flavocoxid.

## Case presentation

**Figure 1:**
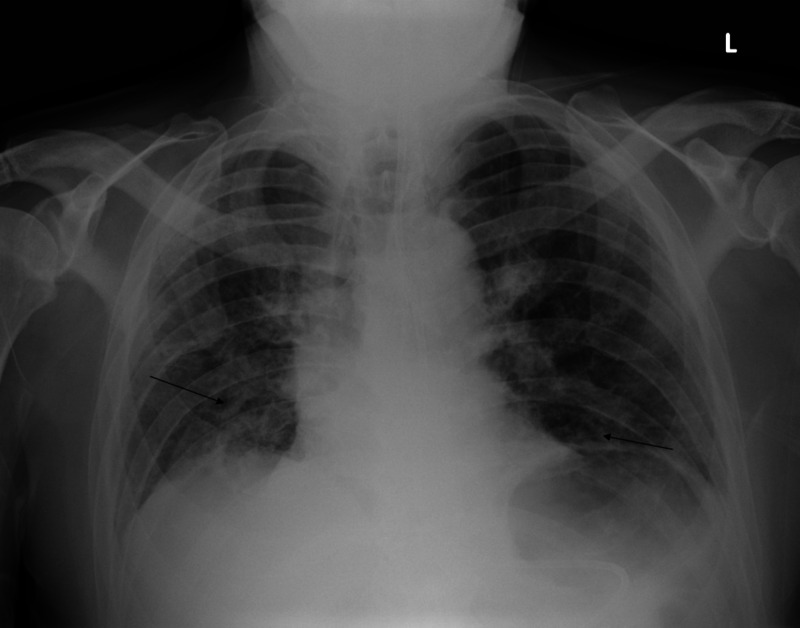
Chest X-ray revealing patchy bibasilar opacities with air bronchograms.

**Figure 2:**
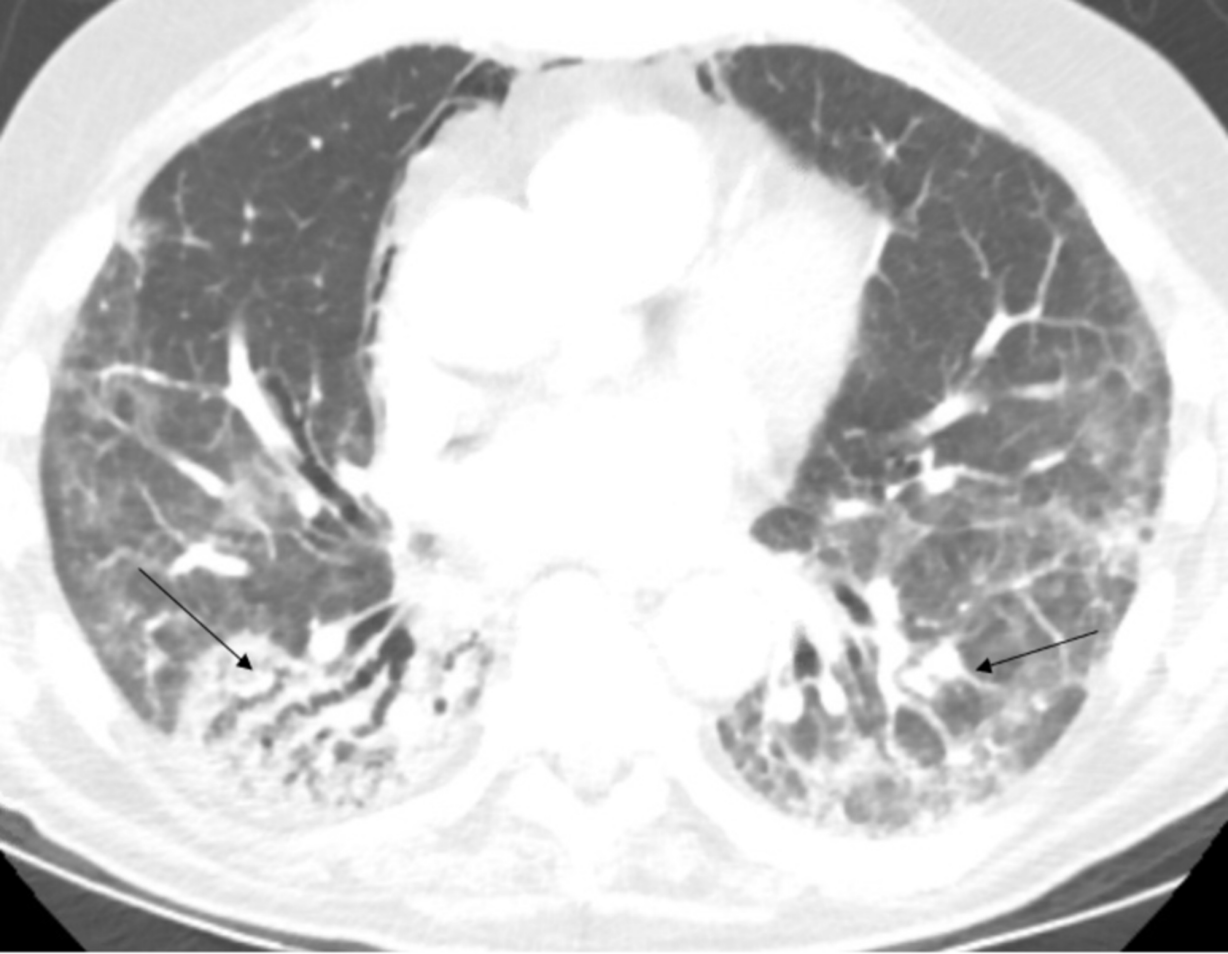
Diffuse bilateral lower lobe consolidation with areas of ground glass opacities.

A 75-year-old male with a past medical history of osteoarthritis and obstructive sleep apnea presented to a tertiary care center with a gradual onset of shortness of breath. His symptoms began two weeks before presentation and was associated with mildly productive cough and fatigue. The patient worked as an engineer and denied occupational exposure. He had no significant medical problems, and was at his usual state of health, including hiking at high altitudes without any symptoms a few months before his initial hospitalization. Presenting vitals were notable for hypoxia with oxygen saturation in the range of 85% on room air as well as tachypnea. Physical examination identified bibasilar rales. Chest X-ray revealed patchy bibasilar opacities with air bronchograms (Figure 1). The patient was admitted for acute hypoxic respiratory failure secondary to multilobar pneumonia and was started on broad-spectrum antibiotics. However, he failed to improve on antibiotics and his respiratory function gradually declined, requiring mechanical ventilation support on the fourth day of his hospitalization. A detailed medication review revealed that the patient was started on flavocoxid for his osteoarthritis of hips and knees two months prior to presentation. The medication was immediately discontinued. CT scan revealed diffuse bilateral lower lobe consolidation with areas of ground glass opacities, septal thickening and bronchiectasis, thought to be secondary to hypersensitivity pneumonitis (Figure 2). He was unable to be weaned from the ventilator, until he was started on IV methylprednisolone and eventually was able to be extubated. His respiratory function never returned to baseline as he became oxygen-dependent after the hospitalization. He was discharged to a pulmonary rehabilitation program on a prolonged steroid tapering regimen.

In the following two years, he continued to have episodes of acute exacerbations of dyspnea and hypoxia with severe exercise intolerance. He was briefly started on mycophenolate mofetil and intermittently with prednisone for six months via the local advanced pulmonary clinic without improvement.

**Figure 3:**
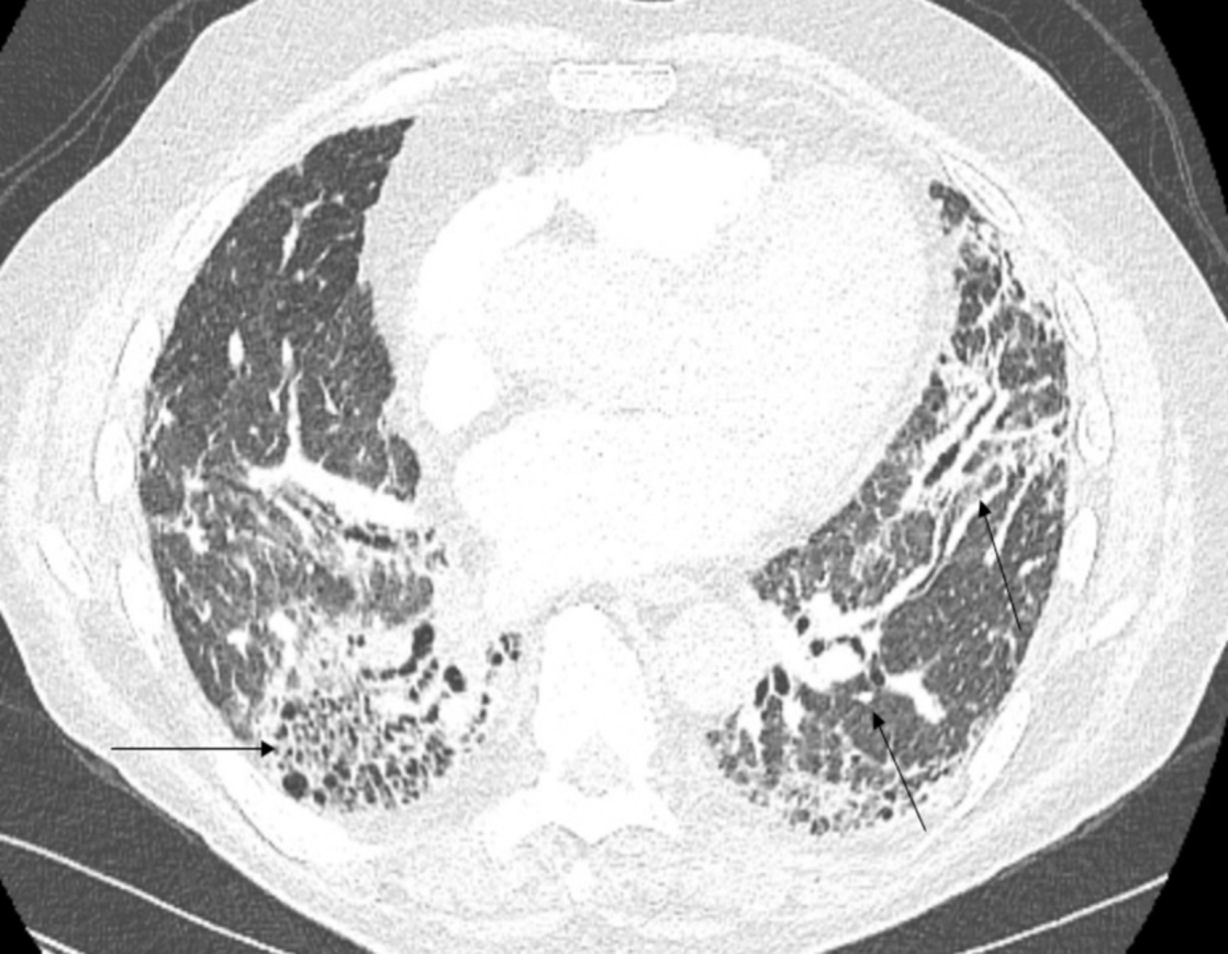
Evidence of bronchiectasis and findings consistent with usual interstitial pneumonia pattern.

Our initial encounter with the patient was approximately two years later when he presented to the same tertiary hospital with shortness of breath and hypoxia. His chest CT scan showed bilateral pulmonary embolism and diffuse "honeycombing" predominantly in the basilar region with subpleural reticular opacities. There was evidence of bronchiectasis and the findings were consistent with usual interstitial pneumonia pattern (Figure 3). Extensive infectious work-up was obtained including viral respiratory panel, Bordetella, Legionella, and Mycoplasma testing among others. Rheumatological work up such as anti-nuclear antibody, rheumatoid factor, and antineutrophil cytoplasmic antibody was negative. He was diagnosed with drug-induced pulmonary fibrosis secondary to flavocoxid use.

## Discussion

Since flavocoxid's introduction into the market, there were reports of hypersensitivity pneumonitis [4-5]. Flavocoxid's toxicity, as reported by patients and physicians from its introduction in 2004 to September 2017, was compiled as a part of post-marketing surveillance. It was found that there were 40 cases of hypersensitivity pneumonitis and few cases of liver injury with jaundice with more than 500,000 prescriptions filled during that time. These adverse events resolved upon flavocoxid discontinuation [4-5]. In our case, flavocoxid-induced hypersensitivity pneumonitis did not improve upon discontinuation of the drug and ultimately progressed to pulmonary fibrosis.

Drug-induced pulmonary fibrosis can be caused by chronic administration of certain drugs such as bleomycin, amiodarone, and cyclophosphamide. The most common symptoms include dyspnea, nonproductive cough, and crackles on examination. It is diagnosed by chest radiography, pulmonary function testing, and high-resolution computed tomography (HRCT) [6]. The presence of usual interstitial pneumonia (UIP) pattern on HRCT is sufficient for diagnosis, and surgical biopsy is generally not needed [7]. In contrast to UIP pattern, in chronic hypersensitivity pneumonitis the HRCT reveals upper lung field predominance of lesions with lobular areas of decreased vascularity and attenuation along with centrilobular nodules. Of note, a retrospective study of 66 patients found that HRCT allows for confident distinction between chronic hypersensitivity pneumonitis and idiopathic pulmonary fibrosis (IPF) in approximately half the cases [8].

Adverse drug reaction is defined as “an appreciably harmful or unpleasant reaction, resulting from an intervention related to the use of a medicinal product, which predicts hazard from future administration and warrants prevention, specific treatment, alteration of dosage regimen or withdrawal of product [9].” A few years ago, causality assessment was completely dependent on expert judgment, but with an increasing need for causality assessment and maintaining uniformity by decreasing the disagreement, several algorithms and tools were developed to have a structured approach.

There are many causality assessment tools that can be used to evaluate the causality between the drug and the adverse effect. The Naranjo probability scale is a widely accepted method for causality assessment in clinical practice as they offer a simple methodology [10].

Using the Naranjo algorithm, we investigated if the adverse event was related to the drug administration (see Table 1). On a scale of -4 (unlikely) and +13 (definite), the patient’s Naranjo probability scale score was found to be +5, as the adverse event appeared after the suspected drug was administered (+2), a lack of alternative cause (+2), and the adverse event -- the development of pulmonary fibrosis -- was confirmed by objective evidence - HRCT (+1). A score of +5 indicates probable cause as the “reaction followed a reasonable temporal sequence after a drug” and “could not be reasonably explained by the known characteristics of the patient’s clinical state.”

We considered the alternate possibility of IPF if his hypersensitivity pneumonitis improved and if he developed pulmonary fibrosis later in life; however, our patient did not get better after discontinuation of the drug, and his pneumonitis gradually progressed to pulmonary fibrosis; hence we attribute this to the adverse effect of the drug.

**Table 1:** Completed Naranjo scale for our case with a score of five, which indicates a probable relationship between flavocoxid (Limbrel) and the development of pulmonary fibrosis.

S.No	Question	Yes	No	Do not know	Score
1	Are there previous conclusive reports on this reaction?	+1	0	0	0
2	Did the adverse event appear after the suspected drug was administered?	+2	-1	0	+2
3	Did the adverse reaction improve when the drug was discontinued or a specific antagonist was administered?	+1	0	0	0
4	Did the adverse event reappear when the drug was re-administered?	+2	-1	0	0
5	Are there alternative causes (other than the drug) that could on their own have caused the reaction?	-1	+2	0	+2
6	Did the reaction reappear when a placebo was given?	-1	+1	0	0
7	Was the drug detected in the blood (or other fluids) in concentrations known to be toxic?	+1	0	0	0
8	Was the reaction more severe when the dose was increased or less severe when the dose was decreased?	+1	0	0	0
9	Did the patient have a similar reaction to the same or similar drugs in any previous exposure?	+1	0	0	0
10	Was the adverse event confirmed by objective evidence?	+1	0	0	+1
		Total score			5

## Conclusions

This case highlights the importance of thorough history taking and detailed medication review. Thorough inquiry about supplements, medical foods, and alternative medications should be an integral part of history. Clinicians should also educate themselves and the patient on potential adverse effects and drug interactions of commonly prescribed medications. Multi-disciplinary rounds involving a pharmacist could be of great benefit in the identification of nascent medications and active monitoring of adverse drug reactions.

## References

[1] Gottlieb D, Kuritzky L (2011). Using the medical food flavocoxid [corrected] in managing osteoarthritis. J Pain Palliat Care Pharmacother.

[2] Morgan SL, Baggott JE, Moreland L (2009). The safety of flavocoxid, a medical food, in the dietary management of knee osteoarthritis. J Med Food.

[3] Altavilla D, Squadrito F, Bitto A (2009). Flavocoxid, a dual inhibitor of cyclooxygenase and 5-lipoxygenase, blunts pro-inflammatory phenotype activation in endotoxin-stimulated macrophages. Br J Pharmacol.

[4] Levy RM (2011). Flavocoxid and hypersensitivity pneumonitis. Chest.

[5] Panduranga V, Atienza J, Kumar A (2013). Hypersensitivity pneumonitis due to flavocoxid: are corticosteroids necessary?. Conn Med.

[6] Daba MH, El-Tahir KE, Al-Arifi MN (2004). Drug-induced pulmonary fibrosis. Saudi Med J.

[7] Raghu G, Collard HR, Egan JJ (2011). An official ATS/ERS/JRS/ALAT statement: idiopathic pulmonary fibrosis: evidence-based guidelines for diagnosis and management. Am J Respir Crit Care Med.

[8] Silva CI, Müller NL, Lynch DA (2008). Chronic hypersensitivity pneumonitis: differentiation from idiopathic pulmonary fibrosis and nonspecific interstitial pneumonia by using thin-section CT. Radiology.

[9] Naidu RP (2013). Causality assessment: a brief insight into practices in pharmaceutical industry. Perspect Clin Res.

[10] Naranjo CA, Busto U, Sellers EM (1981). A method for estimating the probability of adverse drug reactions. Clin Pharmacol Ther.

